# Anti-N-methyl-D-aspartate Receptor Encephalitis

**DOI:** 10.7759/cureus.5192

**Published:** 2019-07-22

**Authors:** Gregory Tanquary, William Fraser, Kaitlin M Bowers

**Affiliations:** 1 Emergency Medicine, OhioHealth Doctors Hospital, Columbus, USA; 2 Emergency Medicine, Hilton Head Hospital, Hilton Head Island, USA

**Keywords:** nmda receptor encephalitis, ovarian teratoma, altered mental status, autoimmune encephalitis, lumbar puncture, encephalitis, immunotherapy

## Abstract

Anti-N-methyl-D-aspartate receptor (NMDA) encephalitis is an underrecognized encephalitis that may be mistaken for a wide variety of mental illnesses and causes of delirium. This syndrome is predominantly present in young females presenting with acute psychotic episodes, autonomic instability, and neurologic abnormalities. It is commonly associated with ovarian teratoma. Our case illustrates anti-NMDA encephalitis presenting in a young female with progressive mental status changes and neurologic abnormalities throughout her emergency department course. We review the investigative approach, diagnostic modalities, and treatment options in patient management. This case emphasizes the need for a high index of suspicion of anti-NMDA receptor encephalitis when approaching a patient with unexplained changes in mentation.

## Introduction

Anti-N-methyl-D-aspartate receptor (NMDA) encephalitis is a rare and potentially deadly syndrome. While little is understood about this disease process, epidemiologic studies suggest that anti-NMDA receptor encephalitis may be the most common encephalitis after acute demyelinating encephalitis such as West Nile, Coxsackie, or herpes simplex [1]. While prevalence rates are difficult to estimate based on the rarity and difficulty in diagnosis of the disease, more than 500 cases have been reported across multiple series [2-4]. This syndrome may only be encountered once in a career, so, it is important for the emergency physician to maintain a high index of suspicion as early diagnosis and treatment leads to improved morbidity and mortality [5].

Most of the present studies on anti-NMDA encephalitis are case reports. The diagnosis and subsequent treatment is challenging for the emergency physician due to its vague presentation coupled with delayed onset of neurologic symptoms. We offer a unique case of the undifferentiated patient presenting with altered mental status.

## Case presentation

A 30-year-old female presented to the emergency department (ED) with a chief complaint of “not feeling right” for the past six days. The patient had previously presented to an urgent care at the request of a co-worker who stated she was “not acting right.” The patient’s only complaints were headache, congestion, and fatigue. She denied any drug or alcohol ingestions. She denied any suicidal ideation or hallucinations. She had no other physical complaints. She endorsed no medical, surgical or psychiatric history. The patient’s initial vital signs were within normal limits.

Upon examination, the patient was alert and oriented without any focal neurological deficits. Her speech was normal. She appeared anxious with an inappropriate affect. She also exhibited abnormal memory and was inattentive. She underwent a typical altered mental status workup that included: urinalysis (UA), complete blood count (CBC), complete metabolic panel (CMP), thyroid stimulating hormone (TSH), Acetaminophen (APAP), Salicylates (ASA), urine drug screen (UDS), urine pregnancy, venous blood gas (VBG), and non-contrast head computed tomography (CT). UA was hazy with moderate leukocyte esterase, five white blood cells (WBCs) and rare bacteria. CBC was within normal limits with WBCs of 8.84 mm^3^, hemoglobin of 13.3 g/dL, hematocrit of 40.4% and platelets of 413 mm^3^. CMP resulted in a sodium of 143 mEq/L, chloride of 98 mEq/L, potassium of 3.5 mEq/L, bicarbonate of 23 mEq/L, creatine of 0.8 mg/dL. TSH was 0.66 μU/mL. APAP and ASA levels were within normal limits. UDS and pregnancy test were both negative. VBG showed a pH of 7.38, pCO2 of 40.6 mmHg and pO2 of 29 mmHg. Head CT showed no acute process. Overall, the patient's laboratory and imaging findings were grossly unremarkable. The patient continued to show signs of inappropriate behavior including perseveration as to why she was in the hospital. She did not exhibit signs that she had capacity to make medical decisions for herself. Given this she was placed on a medical hold in the ED until she could be evaluated by the behavioral health team.

Throughout her emergency department stay her mental status declined. The patient’s speech pattern became more repetitive and her affect more detached. She required reminders multiple times as to the reason for her ED visit. She was redirected by nursing multiple times throughout the night. Furthermore, the patient began to exhibit signs of visual hallucinations and response to external stimuli. The patient’s detachment progressed to uncooperativeness and ultimately required physical and chemical sedation. Her vital signs remained unchanged throughout this time.

**Figure 1:**
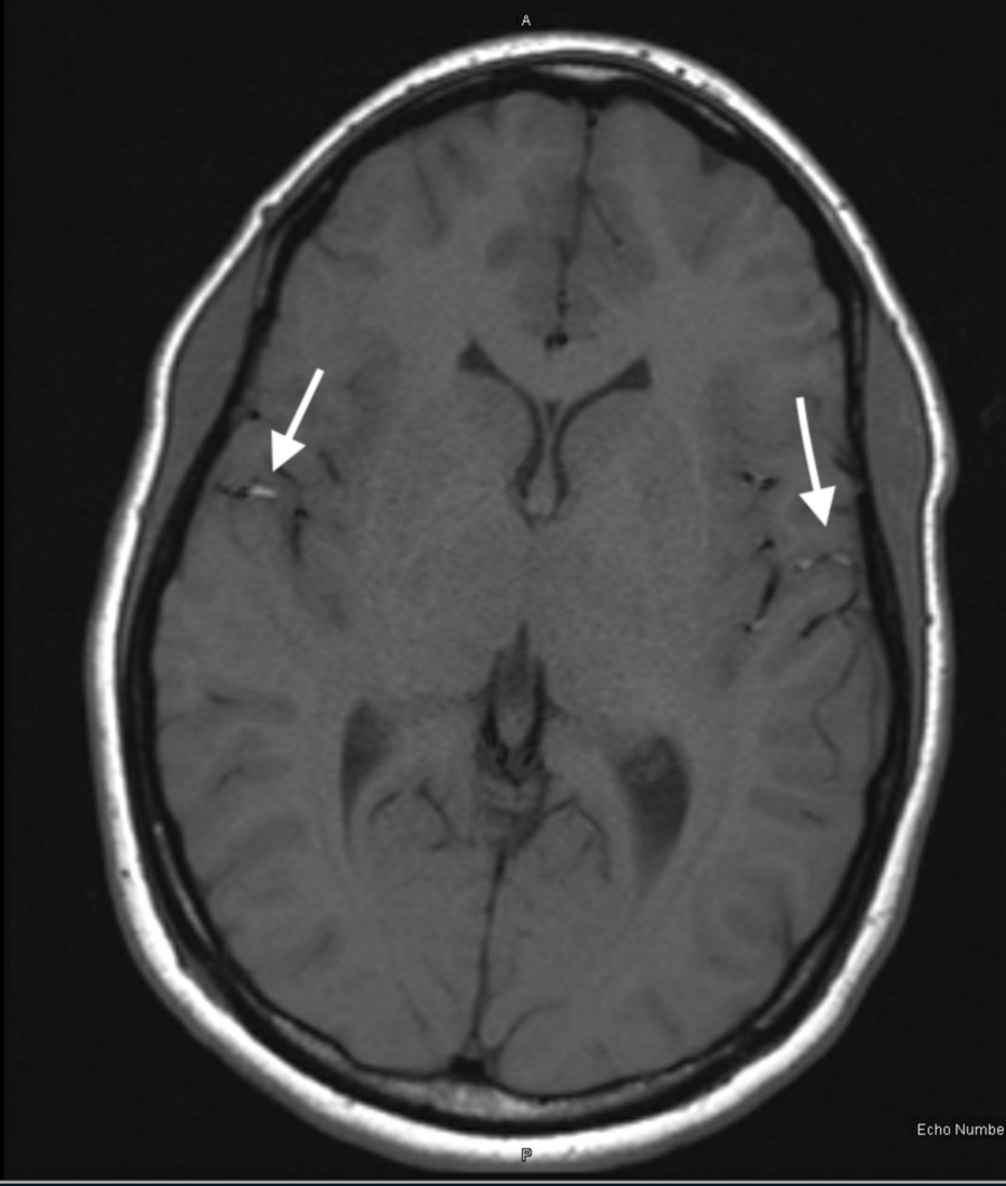
Axial T2 Flair MRI Brain

Approximately 12 hours after her initial presentation she was evaluated by the behavioral health team. They agreed the patient did not have capacity to make her own decisions. Behavioral health was concerned for an organic process as they felt there was no evidence of primary mood, anxiety or psychotic disorder. Thus recommending hospital admission with neurological consultation. Pelvic ultrasound, recommended by neurology, was obtained inpatient and showed signs of a right-sided paraovarian cyst. Magnetic resonance (MR) brain with and without contrast was consistent with linear enhancement on the surface of the brain (Figure 1).

**Figure 2:**
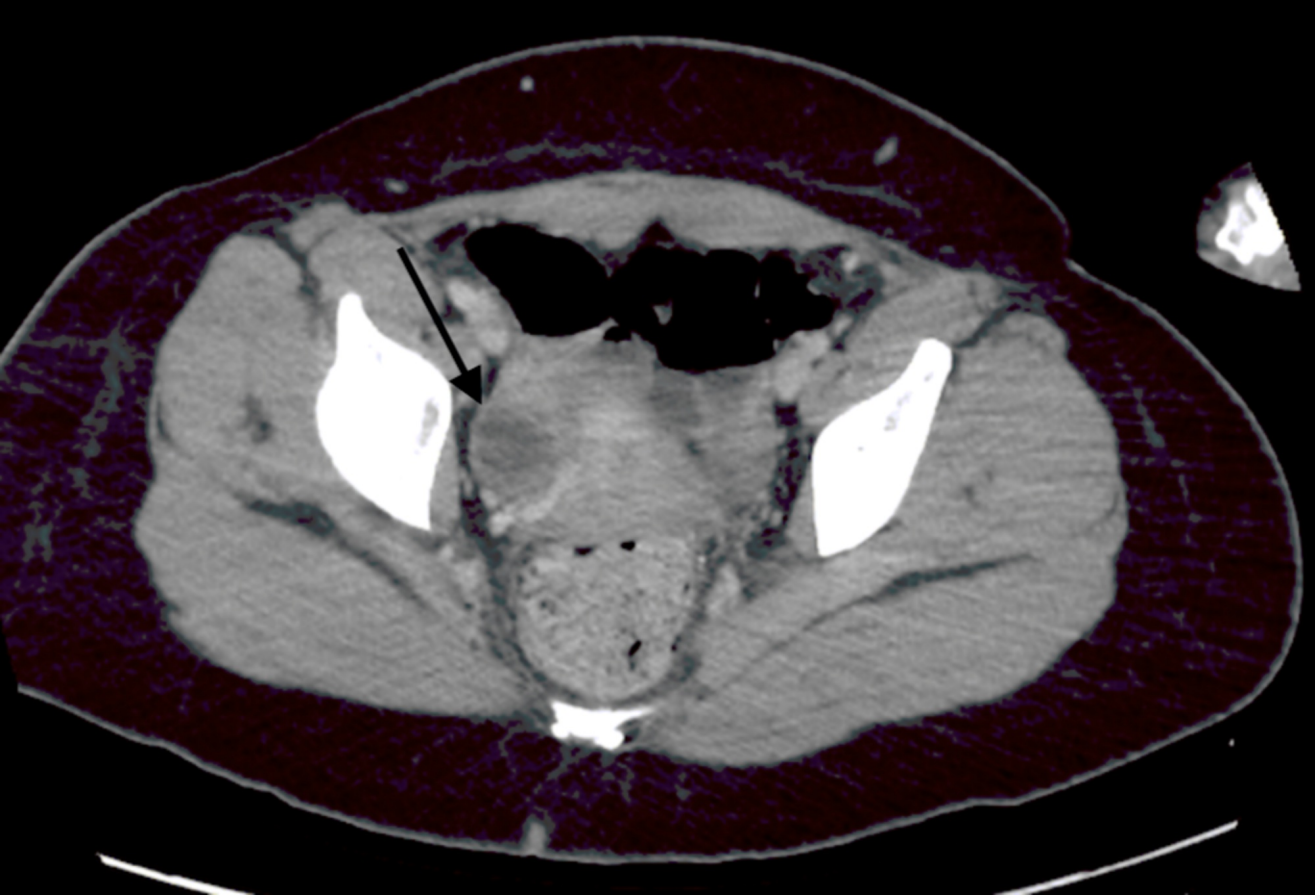
Axial CT Chest Abdomen and Pelvis with IV Contrast

**Figure 3:**
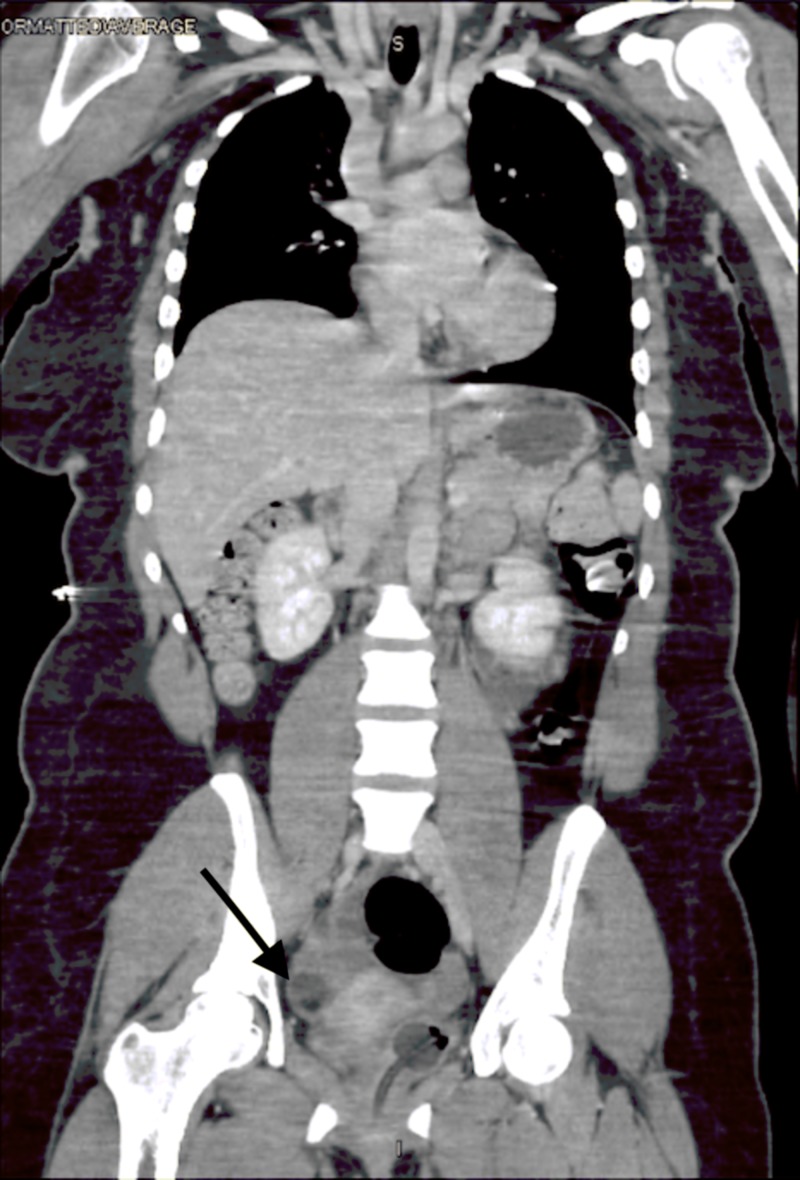
Coronal CT Chest Abdomen and Pelvis with IV Contrast

**Figure 4:**
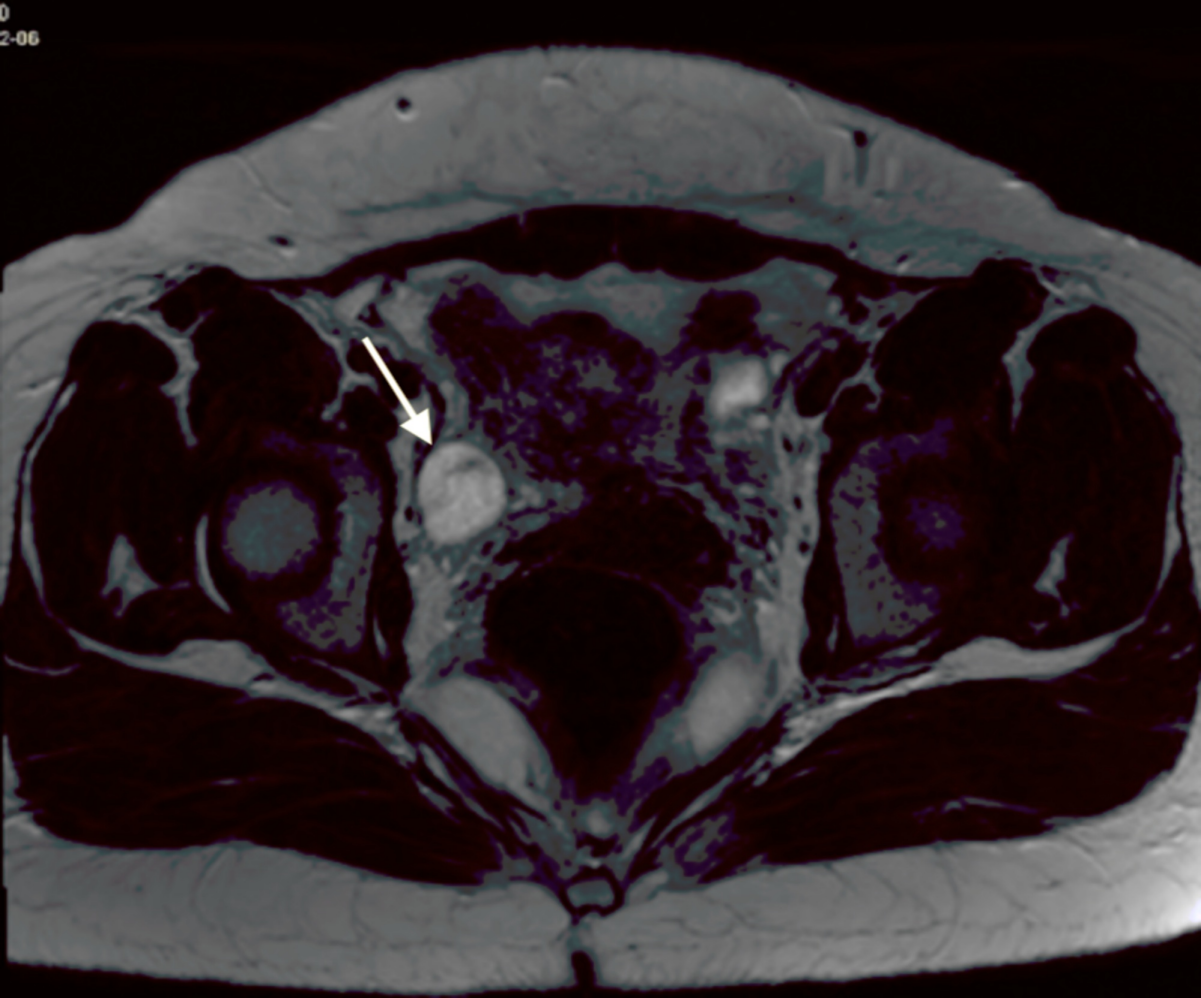
Axial MRI Pelvis with IV Contrast

**Figure 5:**
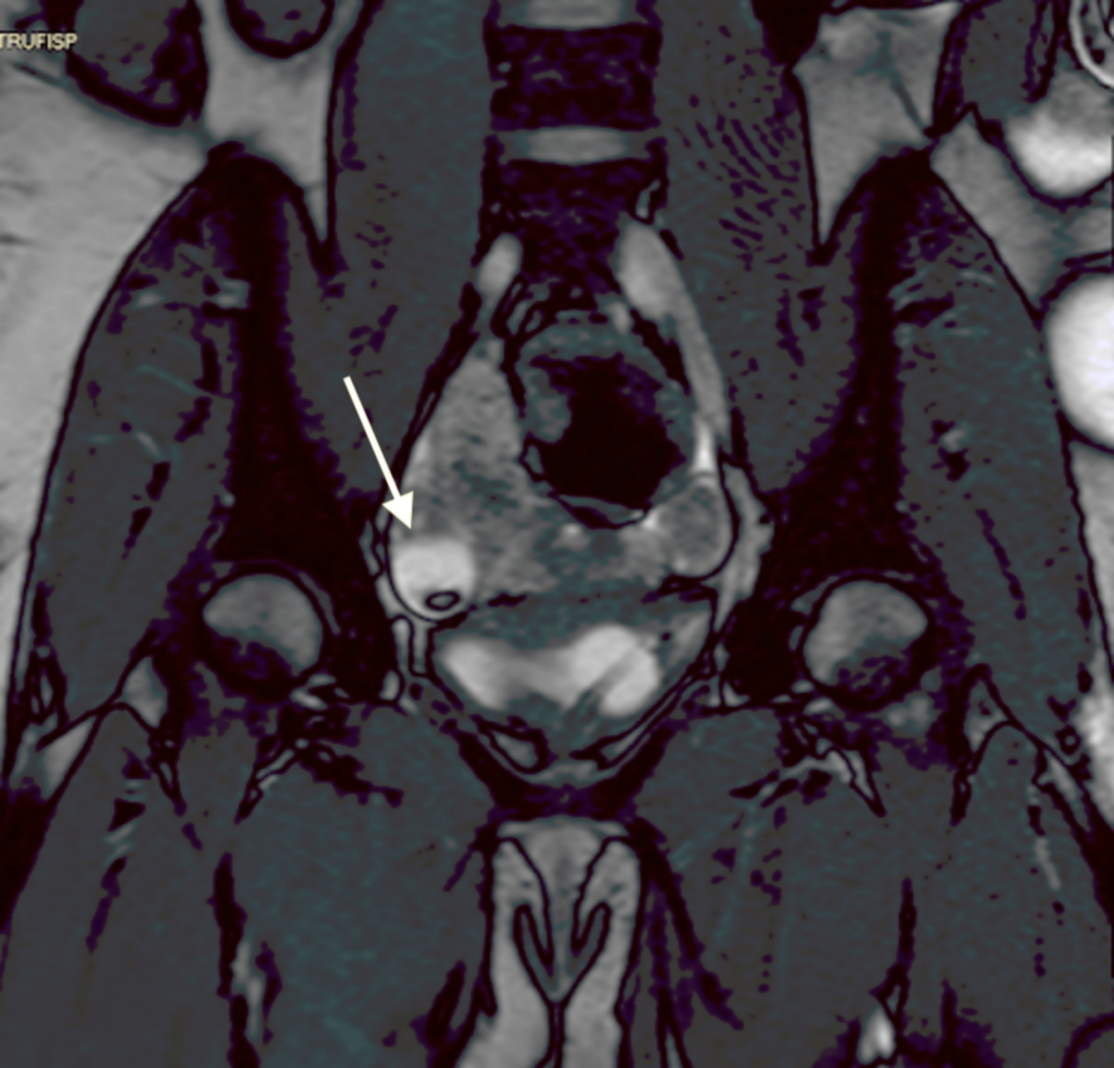
Coronal MRI Pelvis with IV Contrast

Lumbar puncture was performed by the inpatient team, results showed 118 WBCs, 96% lymphocytes, normal glucose, normal protein, gram stain negative, and herpes simplex virus biofire negative. Follow-up CT chest abdomen pelvis (Figures 2, 3) and MR pelvis with contrast (Figures 4, 5) were obtained to further evaluate the pelvic ultrasound findings. Advanced imaging was consistent with a right ovarian dermoid cyst.

The findings on her imaging coupled with her presentation lead the patient to be started on intravenous (IV) steroids, immunoglobulin therapy, and plasma exchange for suspected anti-NMDA receptor encephalitis. On hospital day 11, the patient underwent laparoscopic right oophorectomy for cyst removal. The patient was subsequently intubated on hospital day 13 due to catatonia, decreased responsiveness, and posturing. Hospital day 15 and post-operative day four, the patient developed tachycardia and hypotension. She was found to be profoundly anemic and laboratory studies were consistent with disseminated intravascular coagulopathy. Massive transfusion protocol was initiated and the patient was taken to the operating room (OR) emergently for an exploratory laparotomy. Ligation of inferior right epigastric artery was performed for bleeding source control. The patient returned to the OR two additional times for persistent bleeding and hematoma evacuation. She continued to develop renal failure and required renal replacement therapy. On hospital day 21, the patient returned to OR for complex laparotomy wound closure without complication. On hospital day 25, the patient’s mother requested transfer to quaternary care facility for further evaluation. The patient later succumbed to her disease process.

## Discussion

Encephalitis is an inflammatory condition of the brain with many potential etiologies. There are several classes of encephalitis including paraneoplastic and autoimmune mediated encephalitis. While paraneoplastic encephalopathies are, by definition, cancer-related, autoimmune encephalitis may occur in the presence or absence of cancer. Nomenclature regarding the pathophysiology of anti-NMDA receptor encephalitis can be confusing. Anti-NMDA encephalitis is considered an autoimmune encephalitis, however it is commonly associated with ovarian teratoma as discovered by Dalmau et al. [6]. This makes it possible for anti-NMDA receptor antibody encephalitis to be paraneoplastic and autoimmune mediated simultaneously.

Although uncommon, anti-NMDA antibody receptor encephalitis is one of the best described subgroups of autoimmune encephalopathy. This unique subtype is mediated by immunoglobulin G antibodies against GluN1 subunit of the neuronal NMDA receptors. This results in inflammatory neuronal dysfunction and destruction [7]. Current evidence suggests this neuronal dysfunction can be reversible if recognized and treated early. However, all cases progress to permanent neuronal destruction if left untreated, further emphasizing the importance of early recognition at the index emergency visit.

Signs and symptoms

Patients typically present with vague symptoms such as fever, headache and general fatigue. These symptoms usually progress over the course of one to two weeks. Most patients then progress to developing bizarre behavior, disorientation, confusion, paranoia, or hallucinations [6]. Autonomic instability is usually a late finding and includes decreased consciousness, hypoventilation, seizures, and dyskinesias. According to a case series by Dalmau et al., 76% experienced seizures and 88% suffered from decreased consciousness and akinesis [6].

While literature regarding anti-NMDA receptor encephalitis is mostly limited to case series, commonalities between patients have been discovered. Up to 90% of patients affected by this syndrome are female. Also, unique to this syndrome is the presence of ovarian teratoma, which has been found in 60% of young patients with anti-NMDA encephalitis [6]. The detection of ovarian teratoma is also age dependent.

Older patients diagnosed with anti-NMDA encephalitis have been found to have less severe cases of disease. However, the median time to diagnosis is increased in this age group (eight weeks as opposed to four weeks in young adults) leading to increased morbidity and mortality due to delayed treatment [8]. This delay in diagnosis is thought to be due to absence of prodromal symptoms. Only 26% of patients over age 45 have prodromal symptoms compared to 54% in the younger group [8]. This older subgroup of patients tend to present with memory deficits which leads to a wider differential, making it difficult to diagnose in the early disease state. In this age group, the male to female distribution evens out with 45% of patients over 45 being male. The association with tumors also decreases to only 23%, which are rarely teratomas [8].

Diagnosis

The diagnosis of anti-NMDA receptor encephalitis is difficult and confounded by the nonspecific initial presenting symptoms. Commonly, the diagnosis is not considered until the patient has failed to respond to treatments of more common disease states. The differential in most cases includes infectious encephalitis, neuroleptic malignant syndrome, metabolic disorders, psychiatric disorders, cerebral space-occupying lesions, other autoimmune etiologies and drugs, toxins or withdrawal.

Anti-NMDA receptor encephalitis is typically a diagnosis of exclusion and initial workup should be targeted to exclude other causes of encephalopathy. If initial workup does not reveal the cause of the patient’s encephalopathy, it is important to rule out anti-NMDA receptor encephalitis, especially in the female patient with no known psychiatric history. A classic pitfall is mis-diagnosing these patients with new onset psychiatric illness and initiating antipsychotic therapy. Due to the high morbidity and mortality with delayed diagnosis it is important for the ED physician to complete a thorough workup. Additional workup should include pelvic ultrasound looking for presence of ovarian teratoma given the high association of ovarian teratoma and NMDA encephalitis [6]. Lumbar puncture analyzing cerebrospinal fluid (CSF) should also be obtained in the ED. NR1 and NR2 antibodies should be added to the CSF analysis as they are pathognomonic for diagnosing anti-NMDA receptor encephalitis [9]. Due to prolonged laboratory turnaround times it is prudent to treat for anti-NMDA encephalitis prior to results if there is a high index of suspicion.

Treatment

Treatment for anti-NMDA receptor encephalitis includes immunotherapy and early tumor removal in the setting of paraneoplastic cases. Immunotherapy includes high-dose corticosteroids, IV immunoglobulin, and exchange transfusion. The hospital course for patients with this disease state is often complicated by autonomic instability, catatonia, and end-organ damage [4].

Early diagnosis of the disorder could lead to effective management and better outcomes. According to Dalmau et al., out of 100 cases who underwent treatment less than four months after symptom onset 47% had a full recovery, 28% mild stable neurologic deficits, 18% severe neurologic deficits and 7% expired. This highlights the importance of having anti-NMDA receptor encephalitis on the differential in any case of undifferentiated encephalitis or new onset psychiatric illness in the emergency department, especially in the young female population.

## Conclusions

Anti-NMDA receptor encephalitis is a disease that carries a high mortality and morbidity unless recognized early. Presenting symptoms are typically vague making it difficult to diagnose, especially in the emergency department setting. As seen in our case, it is a common pitfall for patients to be initially misdiagnosed as a new onset psychiatric illness. It is important for the emergency physician to stay vigilant and consider this diagnosis in any young female presenting with bizarre behavior without a history of psychiatric illness. Prior to CSF results, pelvic ultrasound demonstrating ovarian teratoma can be key to early diagnosis and treatment from the emergency department.
